# Preoperative Intravenous Indocyanine Green Injection Demarcates Tumor Border and Adjacent Nerves in Surgical Resection of Posterior Mediastinal Neurogenic Tumors

**DOI:** 10.1093/icvts/ivaf211

**Published:** 2025-12-24

**Authors:** Kongxu Dai, Feng Yang, Yun Li, Jian Zhou

**Affiliations:** Department of Thoracic Surgery, Peking University People’s Hospital, Beijing 100044, China; Thoracic Oncology Institute, Peking University People’s Hospital, Beijing 100044, China; Research Unit of Intelligence Diagnosis and Treatment in Early Non-small Cell Lung Cancer, Chinese Academy of Medical Sciences, 2021RU002, Peking University People’s Hospital, Beijing 100044, China; Department of Thoracic Surgery, Peking University People’s Hospital, Beijing 100044, China; Thoracic Oncology Institute, Peking University People’s Hospital, Beijing 100044, China; Research Unit of Intelligence Diagnosis and Treatment in Early Non-small Cell Lung Cancer, Chinese Academy of Medical Sciences, 2021RU002, Peking University People’s Hospital, Beijing 100044, China; Department of Thoracic Surgery, Peking University People’s Hospital, Beijing 100044, China; Thoracic Oncology Institute, Peking University People’s Hospital, Beijing 100044, China; Research Unit of Intelligence Diagnosis and Treatment in Early Non-small Cell Lung Cancer, Chinese Academy of Medical Sciences, 2021RU002, Peking University People’s Hospital, Beijing 100044, China; Department of Thoracic Surgery, Peking University People’s Hospital, Beijing 100044, China; Thoracic Oncology Institute, Peking University People’s Hospital, Beijing 100044, China; Research Unit of Intelligence Diagnosis and Treatment in Early Non-small Cell Lung Cancer, Chinese Academy of Medical Sciences, 2021RU002, Peking University People’s Hospital, Beijing 100044, China

**Keywords:** posterior mediastinal tumors, indocyanine green, near-infrared fluorescence

## Abstract

**Objectives:**

To assess the application of preoperative indocyanine green (ICG) near-infrared (NIR) fluorescence imaging in video-assisted thoracoscopic surgery (VATS) for posterior mediastinal neurogenic tumors.

**Methods:**

We present a case of a 66-year-old female with a T3-adjacent posterior mediastinal tumor. She received intravenous ICG (5 mg/kg) 24 hours before surgery. Intraoperative NIR imaging was used to identify the tumor and nearby nerves during VATS.

**Results:**

ICG fluorescence clearly delineated the tumor margins and visualized the adjacent sympathetic and intercostal nerves. This guided the complete resection of the tumor, which extended into the intervertebral foramen, with no postoperative complications. Pathology confirmed a schwannoma.

**Discussion:**

ICG NIR imaging offers enhanced intraoperative visualization for complex posterior mediastinal tumors. It aids in distinguishing tumor from normal tissue and identifying key nerves, thereby facilitating complete resection while minimizing the risk of iatrogenic injury. This technique shows promise for managing "dumbbell" tumors via a minimally invasive approach.

**Conclusion:**

Preoperative ICG injection is a useful adjunct in VATS for posterior mediastinal tumors, improving the delineation of tumor borders and neural structures to enhance surgical precision and safety.

## INTRODUCTION

Posterior mediastinal tumours are common thoracic surgical diseases, encompassing neurogenic tumours, lymphomas, and bronchogenic cysts. Surgical resection is the primary treatment.^1^ Although generally not overly complex, some cases with severe adhesions or spinal foramen invasion, known as “dumbbell” tumours, increase surgical difficulty and risk of postoperative complications such as Horner’s syndrome and cerebrospinal fluid leaks.^2^

Previous studies have shown that indocyanine green (ICG) near-infrared (NIR) fluorescence imaging can visualize intracranial neurofibromas. Our centre has demonstrated that ICG NIR fluorescence can visualize sympathetic ganglia and lung tumours, with a preoperative dose of 5 mg/kg administered 24 hours before surgery being widely applied.^3^

To date, no studies have reported the use of ICG NIR imaging in video-assisted thoracoscopic surgery (VATS) for resection of posterior mediastinal tumours. We report the initial application of this method in one patient’s surgery.

## CASE REPORT

**Figure 1.**
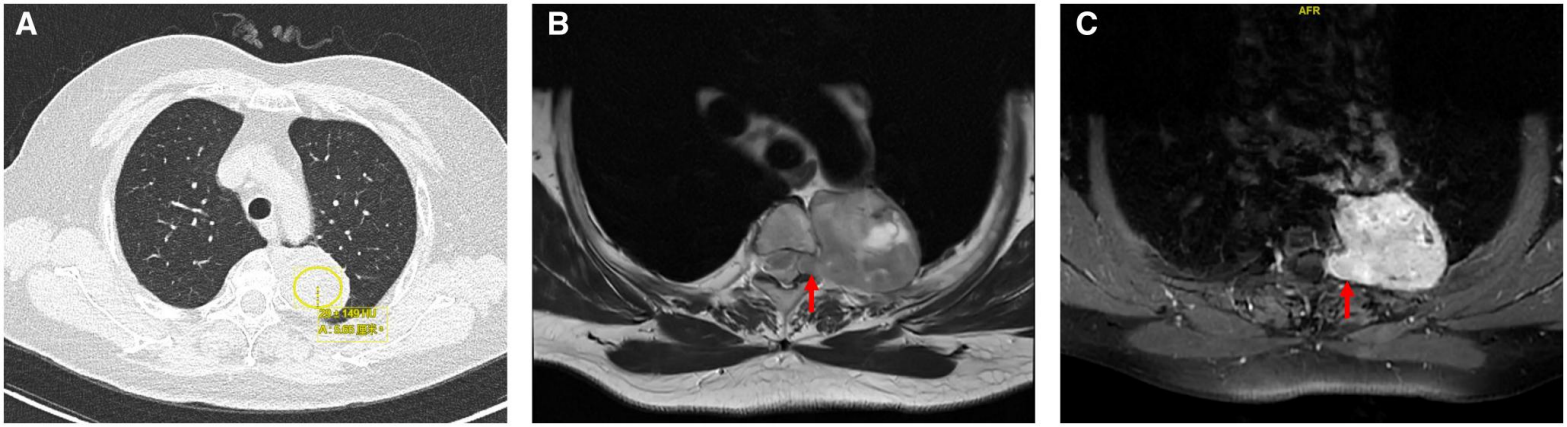
Preoperative Chest CT (A) Reveals a Soft Tissue Mass in the Posterior Upper Mediastinum, Adjacent to the Left Side of the T3 Vertebral Body, Measuring 5.5 × 3.9 cm with a CT Value of 29 ± 149 HU. Enhanced MRI (T1WI B and T2WI C) Demonstrates a Nodular Protrusion (Red Arrow) Extending from the Left T3/4 Intervertebral Foramen, Leading to Foraminal Stenosis and Thickening with Enhancement of the Adjacent Left Pleura

**Figure 2.**
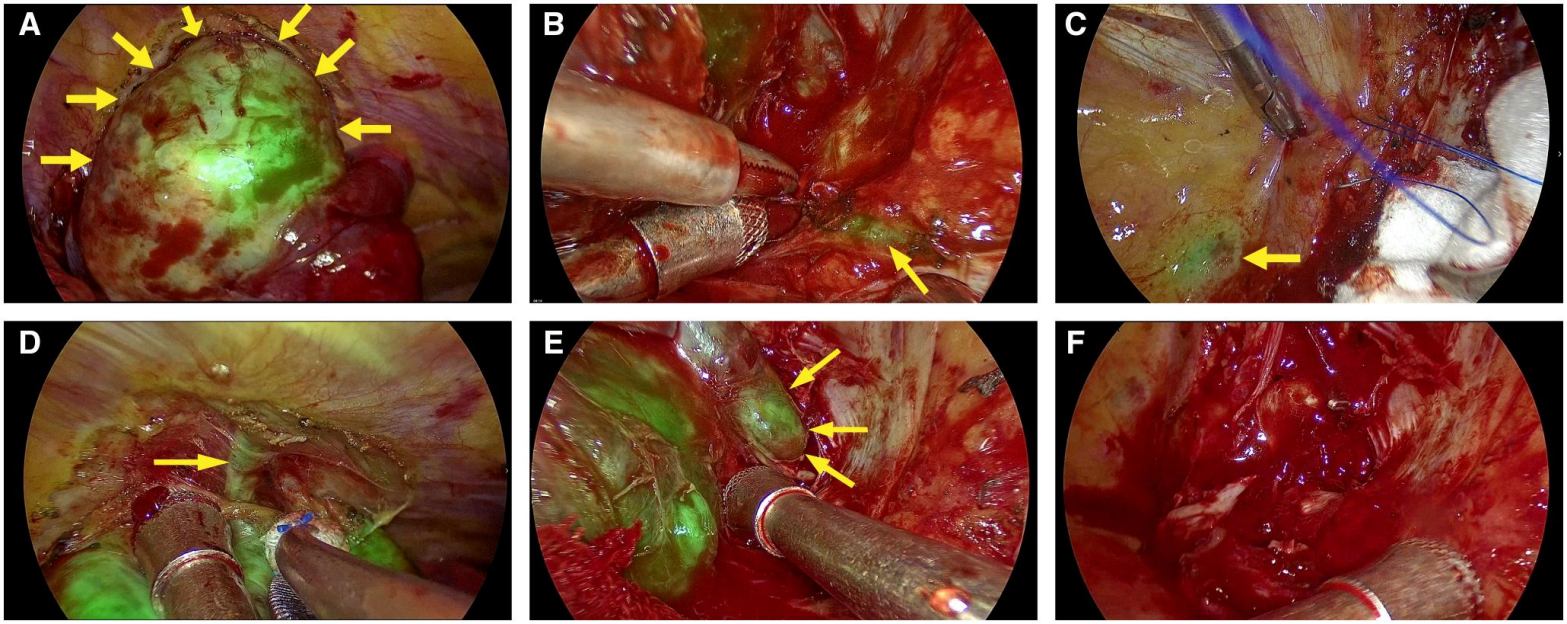
Surgical Procedure. (A) After Opening the Tumour Capsule, Fluorescence Imaging Clearly Visualized the Tumour with Well-Defined Margins. (B) During Dissection of the Lower Pole, the Sympathetic Nerve along the Paravertebral Region Was Visible under Fluorescence. (C) The Sympathetic Ganglion Adjacent to the Upper Pole near the Thoracic Spine Was Identified Intraoperatively. (D) Blunt Dissection Revealed Adhesions Between the Tumour and Chest Wall, with a Distal Connection to the Intercostal Nerve Sheath, Which was Then Transected. (E) Fluorescence Imaging Clearly Demonstrated Tumour Extension into the Intervertebral Foramen. (F) After Complete Resection, the Exposed Intervertebral Foramen and Clear Cerebrospinal Fluid were Observed. Hemostasis Was Achieved Using Hemostatic Material and Sutures

A 66-year-old female with a history of spinal issues presented with left thoracic pain. Her height, weight, and BMI were 150 cm, 54 kg, and 24 kg/m^2^, respectively. Hospitalization revealed a large mass adjacent to the T3 vertebral body on chest CT and mediastinal MRI (**Figure 1**), measuring approximately 5.5 × 3.9 cm, with enlargement of the left T3-4 intervertebral foramen, indicating tumour invasion. After informed consent, she received an intravenous injection of ICG at a dose of 5 mg/kg 24 hours preoperatively. The mass was completely resected surgically (**Figure 2**, **Video 1**). Postoperatively, no significant complications were observed, and pathology confirmed the diagnosis of schwannoma. Immunohistochemical results were as follows: CK(–), Vimentin(+), Resmin(–), SMA(–), S-100(+), β-catenin(+), CD34(+) for vessels, pan-TRK(partially+), STAT6(+), MUC4(-), Sox-10(+), H3K27me3(+), Ki-67(20%+) (**Figure S1**).

## DISCUSSION

Indocyanine green has been widely used in thoracic surgery (**Table S1**). We preliminarily explore the application of ICG near-infrared fluorescent imaging in VATS for posterior mediastinal tumours. In this case, ICG near-infrared fluorescent imaging clearly visualized the tumour boundaries with surrounding normal tissues during surgery, and also provided good imaging of the sympathetic and intercostal nerves, which helped to avoid damage to these vital structures and facilitated complete tumour resection.^3^ Additional cases from our center (**Table S2 Figures S2–4**) further demonstrate the utility of ICG near-infrared fluorescence imaging as an adjunct in posterior mediastinal tumour resection.

For thoracic neurogenic tumours with an intraspinal component, also known as “dumbbell” tumours, which account for approximately 10% of mediastinal neurogenic tumour cases,^2^^,^^4^ a multidisciplinary approach using combined minimally invasive laminectomy and VATS resection is often recommended,^5^ carried out by both thoracic and neurosurgeons. Through this case, we demonstrate that for complex tumours with similar conditions, VATS resection aided by ICG imaging can be performed independently to completely remove the tumour from the intervertebral foramen. This reduces surgical trauma to the patient, decreases the incidence of postoperative complications such as cerebrospinal fluid leakage and bleeding, and shortens hospital stays and treatment costs.

## STRENGTHS AND LIMITATIONS

Our study pioneered the use of ICG NIR fluorescence imaging in posterior mediastinal tumour surgery. The technique was particularly beneficial in complex “dumbbell-shaped” tumours, enabling precise tumour delineation from normal tissue and visualization of critical nerves, such as sympathetic and intercostal, to prevent iatrogenic damage. Despite this, the utility of fluorescence imaging may be limited in straightforward posterior mediastinal tumour resections.

## CONCLUSION

We demonstrate that ICG near-infrared fluorescent imaging can better visualize tumour boundaries in posterior mediastinal tumour surgery. It assists operators in clearly completing tumour separation and resection in various complex situations and clearly shows the course and distribution of sympathetic and intercostal nerves, effectively reducing intraoperative nerve injury.

## Supplementary Material

ivaf211_Supplementary_Data

## Data Availability

All the data and surgical videos in the present manuscript are available from the corresponding author upon reasonable request.
