# Supplementary material for: Preoperative Intravenous Indocyanine Green Injection Demarcates Tumor Border and Adjacent Nerves in Surgical Resection of Posterior Mediastinal Neurogenic Tumors
Source: Interdiscip Cardiovasc Thorac Surg. 2025 Dec 24;41(1):ivaf211. doi: 10.1093/icvts/ivaf211 (PMC12957939; doi:10.1093/icvts/ivaf211)
Supplement: ivaf211_Supplementary_Data [file ivaf211_Supplementary_Data.zip › Supplementary Material.docx]

**Supplementary Figure 1. IHC image of this case**


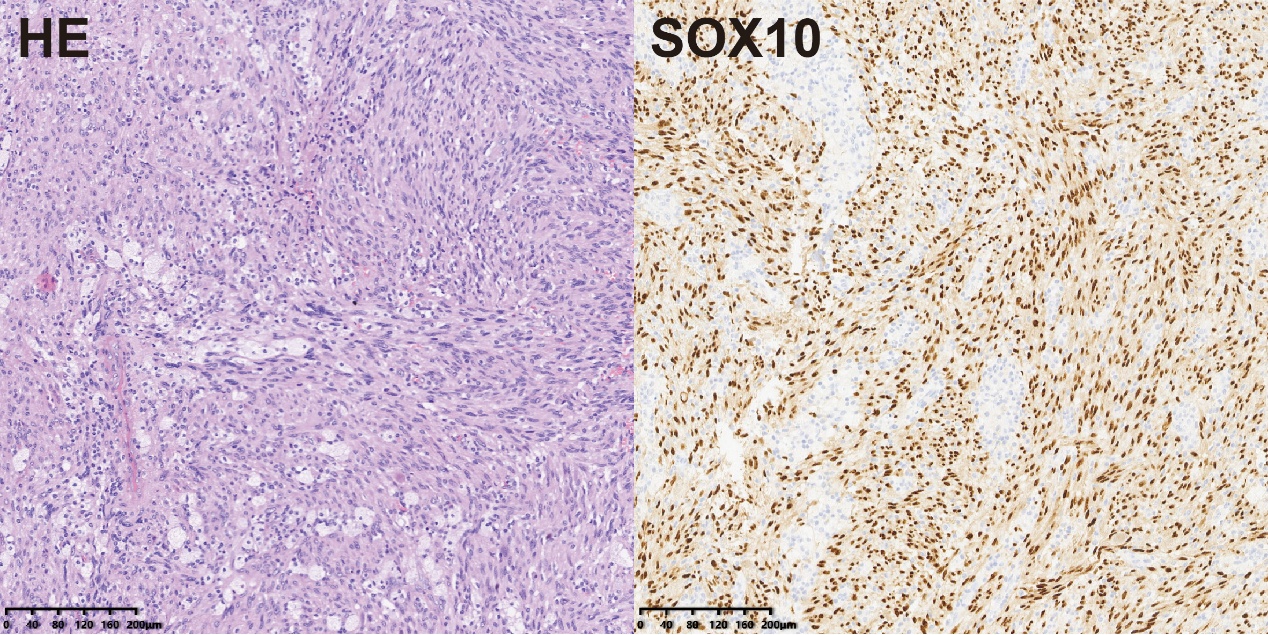


**Supplementary Table 1. The table of similar work**

| Title | Number of Cases | Year | Outcomes |
| --- | --- | --- | --- |
| Thoracoscopic Indocyanine Green Near-Infrared Fluorescence for Thoracic Sympathetic Ganglions | 1 | 2016 | This is the first report about fluorescent imaging of sympathetic ganglions, and this new finding may possibly benefit the sympathectomy of palmar hyperhidrosis. |
| Clinical application of near-infrared thoracoscope with indocyanine green in video-assisted thoracoscopic bullectomy | 2 | 2016 | NIR thoracoscope with intravenous ICG is a safe, accurate and real-time method to detect bullous lesions of lung tissue difficult to be found under normal light in human subjects. |
| Near-infrared fluorescence-guided thoracoscopic surgical intervention for postoperative chylothorax | 4 | 2018 | Near-infrared fluorescence imaging with ICG provided highly sensitive and real-time imaging of the TD in VATS intervention for chylothorax in humans. |
| Case Report: The Second Near-Infrared Window Indocyanine Green Angiography in Giant Mediastinal Tumor Resection | 1 | 2022 | Finally, NIR-II ICG angiography shows the clear location and course of the vessels, which can help surgeons reduce unnecessary blood vessel injury and increase the safety of mediastinal tumor resection. |
| Case report: video-assisted thoracoscopic surgery for pulmonary arteriovenous malformation using near-infrared fluorescence with indocyanine green | 2 | 2023 | We are the first to report the use of such an approach in delineating the margin of vascular malformation with high contrast, and this new finding may help minimize the damage to lung function in PAVM treatment. |

**Supplementary Table 2.The table of prior similar cases**

| Patient | Pathological findings | Postoperative hospital stay(Day) | Surgical complications | Surgical procedure |
| --- | --- | --- | --- | --- |
| A | Schwannoma | 3 | no | Supplementary Figure 2 |
| B | Bronchogenic cyst | 4 | no | Supplementary Figure 3 |
| C | Schwannoma | 4 | no | Supplementary Figure 4 |


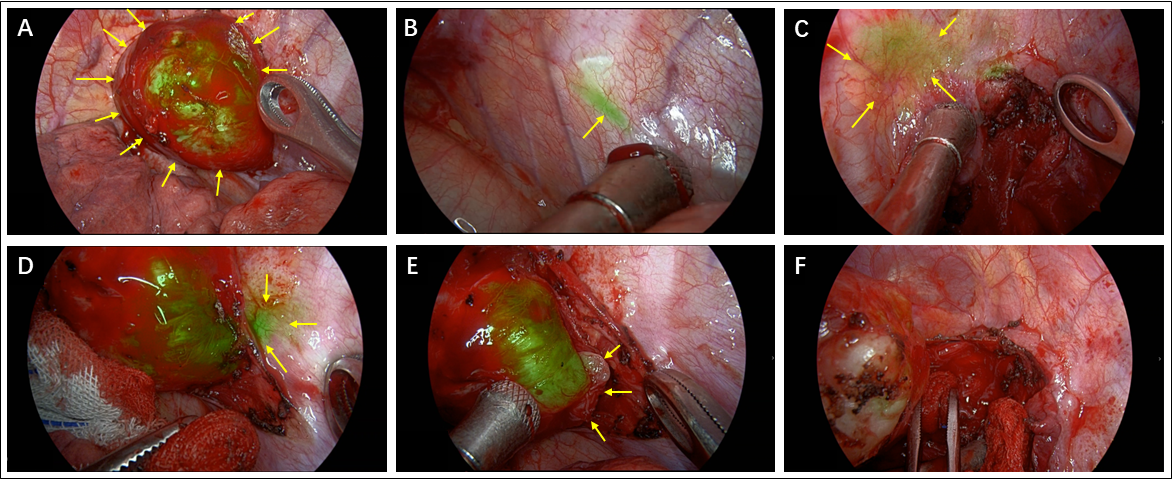


**Supplementary Figure 2.** Surgical procedure for Patient A: (A)Thoracoscopic exploration revealed the mass located at the left upper mediastinum, beside the spine, at the level of the 1st and 2nd intercostal spaces. After opening the mass capsule, the fluorescence imaging showed a clear demarcation of the tumor from the surrounding tissues. (B)Further exploration revealed the sympathetic chain running along the spine. (C)During the procedure, the sympathetic ganglion was visible on the upper pole of the mass under fluorescence imaging. (D)After further opening of the mass capsule, the intercostal nerve was visible. (E)After complete dissection, the mass was found to be connected to the intercostal nerve. (F)The intercostal nerve sheath was divided, and the mass was completely separated.


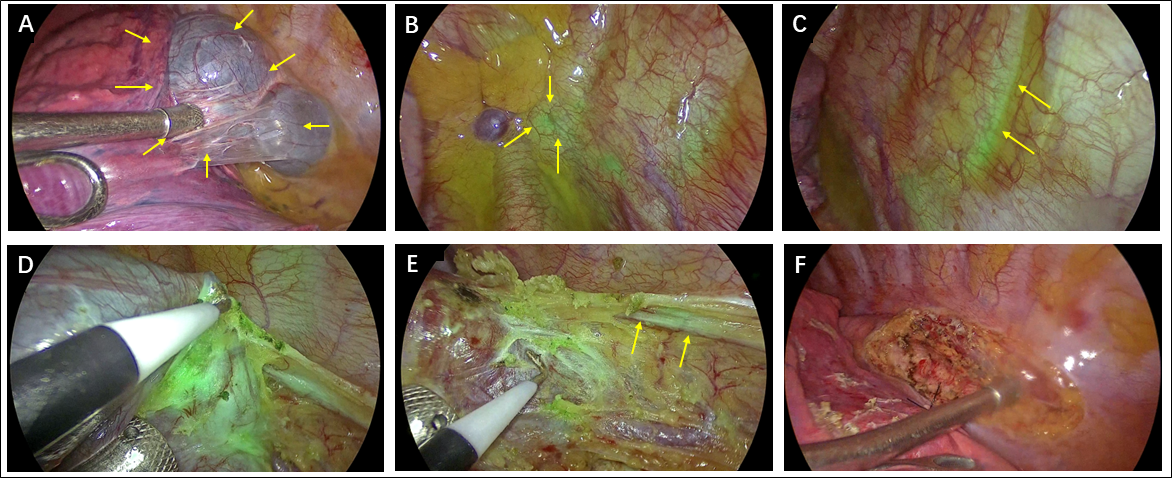


**Supplementary Figure 3.** Surgical procedure for Patient B. (A) Thoracoscopic exploration revealed mild adhesion between the mass and surrounding lung tissue. (B) The superior aspect of the mass showed a fluorescently imaged sympathetic ganglion. (C) The inferior aspect demonstrated a fluorescently imaged intercostal nerve. (D) After opening the mass capsule, the fluorescently imaged mass exhibited clear boundaries from adjacent tissues. (E) During mass dissection, the fluorescently imaged sympathetic nerve was visualized and successfully preserved. (F) Complete mass resection was achieved.


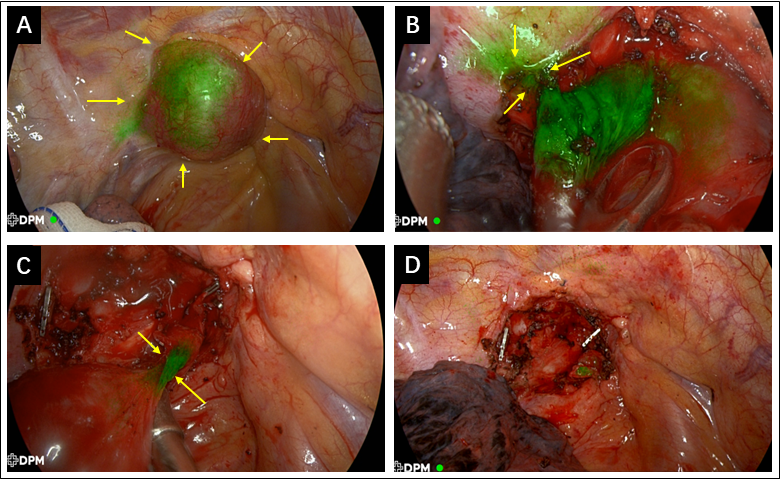


**Supplementary Figure 4.** Surgical procedure for Patient C.(A) Thoracoscopic exploration showed clear boundaries between the fluorescently imaged mass and surrounding tissues.(B) The superior pole of the mass was connected to the sympathetic nerve under fluorescence imaging.(C) During dissection, the inferior pole of the mass was also found attached to the sympathetic nerve.(D) The mass was completely resected.

****Supplementary Materials: CARE Checklist****

**Title:** [Preoperative Intravenous Indocyanine Green Injection Demarcates Tumor Border and Adjacent Nerves in Surgical Resection of Posterior Mediastinal Neurogenic Tumors With a Video Presentation]

**Abstract:** [Surgical resection of posterior mediastinal tumors may lead to postoperative complications such as Horner's syndrome and cerebrospinal fluid leaks. Previous studies have shown that indocyanine green (ICG) near-infrared (NIR) fluorescence can visualize sympathetic ganglia and various tumors. We present the novel use of ICG NIR fluorescence imaging in video-assisted thoracoscopic surgery (VATS) for the resection of posterior mediastinal tumors. A 66-year-old female patient was diagnosed with a posterior mediastinal tumor adjacent to the T3 vertebra, measuring 5.5×3.9 cm. Preoperative administration of ICG at a dose of 5 mg/kg facilitated complete resection of the tumor without postoperative complications. This case illustrates the potential of ICG NIR imaging to enhance the safety and efficacy of VATS for complex posterior mediastinal tumors]

**Introduction:**

- Background: [Posterior mediastinal tumors are common thoracic surgical diseases, encompassing neurogenic tumors, lymphomas, and bronchogenic cysts. Surgical resection is the primary treatment1. Although generally not overly complex, some cases with severe adhesions or spinal foramen invasion, known as "dumbbell" tumors, increase surgical difficulty and risk of postoperative complications such as Horner's syndrome and cerebrospinal fluid leaks]

- Rationale: [Previous studies have shown that indocyanine green (ICG) near-infrared (NIR) fluorescence imaging can visualize intracranial neurofibromas. Our center has demonstrated that ICG NIR fluorescence can visualize sympathetic ganglia and lung tumors, with a preoperative dose of 5mg/kg administered 24 hours prior to surgery being widely applied.

To date, no studies have reported the use of ICG NIR imaging in video-assisted thoracoscopic surgery (VATS) for resection of posterior mediastinal tumors. We report the initial application of this method in one patient’s surgery]

**Methods:**

- Study Design: [Experimental Studies]

- Participants: [Treatment team]

- Interventions: [Administer indocyanine green intravenously to the patient at a dose of 5 mg/kg,24 hours before surgery]

- Outcomes: [The assistance of ICG fluorescence imaging in surgery]

**Results:**

- Participant Flow: [Include one patient in the study]

- Recruitment: [Patients with posterior mediastinal tumors who are scheduled for surgery and are willing to receive indocyanine green injections]

- Baseline Characteristics: [Posterior mediastinal tumor]

- Interventions Received: [Administer indocyanine green intravenously to patients at a dose of 5 mg/kg, 24 hours before surgery, and perform the operation using a thoracoscopic fluorescence lens]

- Outcomes and Estimation: [The tumor was completely removed, and surrounding tissues were protected from damage]

**Discussion:**

- Limitations: [Complete resection of posterior mediastinal tumors is usually straightforward, the value of fluorescence imaging may be limited]

- Interpretation: [ICG fluorescence imaging helps to distinguish tumors, nerves and surrounding normal tissues during posterior mediastinal tumor surgery, reducing surgical damage and complications in complex procedures]

- Generalizability: [ICG fluorescence imaging can be used in any surgery involving posterior mediastinal tumors]

**Other Relevant Sections:**

- Registration: [Not applicable]

- Ethical Approval: [Informed consent was obtained from all individual participants included in the study. The consent process was explained to the participants in a language they understood, and they were given the opportunity to ask questions before providing their consent. Consent was documented in writing.]

- Sources of Funding: [This work was supported by the National Natural Science Foundation of China (Grant No. 92259303) and the CAMS Innovation Fund for Medical Sciences (CIFMS) (Grant No. 2021-I2M-5-002).]
